# Unraveling the Etiology of Dilated Cardiomyopathy through Differential miRNA–mRNA Interactome

**DOI:** 10.3390/biom14050524

**Published:** 2024-04-27

**Authors:** Fernando Bonet, Francisco Hernandez-Torres, Mónica Ramos-Sánchez, Maribel Quezada-Feijoo, Aníbal Bermúdez-García, Tomás Daroca, Elena Alonso-Villa, Carlos García-Padilla, Alipio Mangas, Rocio Toro

**Affiliations:** 1 Medicine Department, School of Medicine, University of Cádiz (UCA), 11003 Cádiz, Spain; fbonetmartinez@gmail.com (F.B.); elena.alonso@gm.uca.es (E.A.-V.); alipio.mangas@uca.es (A.M.); 2 Research Unit, Biomedical Research and Innovation Institute of Cádiz (INiBICA), Puerta del Mar University Hospital, 11009 Cádiz, Spain; 3 Department of Biochemistry and Molecular Biology III and Immunology, Faculty of Medicine, University of Granada, 18016 Granada, Spain; 4 Cardiology Department, Central de la Cruz Roja Hospital, 28003 Madrid, Spain; monica.ramos81@gmail.com (M.R.-S.); maribelquezada2000@gmail.com (M.Q.-F.); 5 Medicine Department, School of Medicine, Alfonso X EL Sabio University, 28691 Madrid, Spain; 6 Cardiovascular Surgery Department, Puerta del Mar University Hospital, 11009 Cádiz, Spainpapasufrido@yahoo.es (T.D.); 7 Department of Experimental Biology, University of Jaen, 23071 Jaen, Spain; cgpadill@ujaen.es; 8 Internal Medicine Department, Puerta del Mar University Hospital, 11009 Cádiz, Spain

**Keywords:** dilated cardiomyopathy, ischemic cardiomyopathy, volume overload, microRNA, etiology, RNA sequencing

## Abstract

Dilated cardiomyopathy (DCM) encompasses various acquired or genetic diseases sharing a common phenotype. The understanding of pathogenetic mechanisms and the determination of the functional effects of each etiology may allow for tailoring different therapeutic strategies. MicroRNAs (miRNAs) have emerged as key regulators in cardiovascular diseases, including DCM. However, their specific roles in different DCM etiologies remain elusive. Here, we applied mRNA-seq and miRNA-seq to identify the gene and miRNA signature from myocardial biopsies from four patients with DCM caused by volume overload (VCM) and four with ischemic DCM (ICM). Gene Ontology (GO) and Kyoto Encyclopedia of Genes and Genomes (KEGG) enrichment analysis were used for differentially expressed genes (DEGs). The miRNA–mRNA interactions were identified by Pearson correlation analysis and miRNA target-prediction programs. mRNA-seq and miRNA-seq were validated by qRT-PCR and miRNA–mRNA interactions were validated by luciferase assays. We found 112 mRNAs and five miRNAs dysregulated in VCM vs. ICM. DEGs were positively enriched for pathways related to the extracellular matrix (ECM), mitochondrial respiration, cardiac muscle contraction, and fatty acid metabolism in VCM vs. ICM and negatively enriched for immune-response-related pathways, JAK-STAT, and NF-kappa B signaling. We identified four pairs of negatively correlated miRNA–mRNA: miR-218-5p-*DDX6*, miR-218-5p-*TTC39C*, miR-218-5p-*SEMA4A*, and miR-494-3p-*SGMS2*. Our study revealed novel miRNA–mRNA interaction networks and signaling pathways for VCM and ICM, providing novel insights into the development of these DCM etiologies.

## 1. Introduction

Dilated cardiomyopathy (DCM) is characterized by an enlarged and dysfunctional left ventricle [1] that encompasses various etiological causes [2]. Globally, this condition is the most common cause of heart failure (HF) and heart transplantation [3]. Ischemic cardiomyopathy (ICM) is one of the most common types of DCM. Although ICM and DCM can lead to similar symptoms of HF, evidence suggests that distinct DCM etiologies may produce different structural and/or functional phenotypes and may respond differently to therapy [4,5,6]. Aortic regurgitation is a valve disease characterized by the inadequate closure of the aortic valve during diastole, resulting in reverse blood flow from the aorta to the left ventricle [7]. Chronic valve regurgitation causes left-ventricle volume overload, leading to a left-ventricle dilatation that remains silent for a long period of time [8]. However, AR patients suddenly progress towards symptoms and HF after this long stable period [9]. Despite the worse prognosis associated with HF due to ICM, current therapies are relatively indifferent to the disease’s etiology. This reflects an incomplete understanding of the biological mechanisms contributing to HF. Hence, a better understanding of the basic pathological mechanisms of distinct DCM etiologies could provide new insight into diverse mechanisms and pave the way for new precision medicine strategies.

MicroRNA (miRNAs) are small endogenous non-coding RNAs that negatively regulate gene expression at the posttranscriptional level. Mechanistic studies have indicated that miRNAs regulate mRNA expression by partially or complementary binding to the complementary sites in the 3′-untranslated regions (3′UTR), inducing mRNA degradation and translational repression [10]. A single miRNA can target a cluster of protein-coding genes, and a single gene can be targeted by many miRNAs [11,12]. miRNAs show unique expression patterns in different cells, tissues, developmental stages, and disease stages. Their influence on gene regulation is key to understanding biological processes in complex diseases [13]. In this regard, an increasing number of studies have demonstrated that miRNAs play a crucial role in various DCM etiologies and have been suggested as potential therapeutic targets [14,15,16,17]. In addition, it has been shown that specific miRNA expression profiles exist for cardiomyopathies of diverse etiologies [18,19,20,21,22].

Prior studies have demonstrated distinct transcriptome signatures between ICM and DCM hearts by RNA-seq [23,24]. However, the differential miRNA and mRNA pattern expression between different DCM etiologies to distinguish etiology-specific molecular pathways, particularly in human tissue models, are scarce. Li et al. analyzed the paired miRNA–mRNA expression profiles of ICM and non-ICM from data downloaded from gene-expression and hybridization-array data repositories (GEO ID: GSE46224) [25]. However, some patients in both cohorts also presented with valve diseases such as pulmonic valve regurgitation, mitral valve regurgitation, AR, and tricuspid valve regurgitation.

In the present study, we aim to determine the mechanisms of miRNA regulation in two distinct DCM etiologies. For this purpose, we used RNA sequencing (RNA-seq) and small RNA-seq to identify etiology-specific transcriptome signatures in myocardial biopsies from volume overload DCM (VCM) and ICM patients without any valve disease. Furthermore, we analyzed the miRNA–mRNA interactome in order to reveal a previously unknown pathway regulating VCM and ICM progression.

## 2. Materials and Methods

### 2.1. Study Population

The study was carried out on myocardial samples from patients undergoing surgery at the Puerta del Mar, Cádiz University Hospital, Spain, after obtaining ethical authorization (133/2019). The population was distributed into two groups: volume overload cardiomyopathy, VCM (n = 9), and ICM (n = 6). The ICM cohort included patients with a history of acute myocardial infarction or significant coronary-artery stenosis (left main artery greater than 50% or any major coronary artery greater than 75%) [26]. The VCM cohort included patients with severe AR that fulfilled the criteria for aortic valve replacement [9,27]. The etiology was determined by two independent cardiologists. None of the patients had familial DCM criteria, additional cardiovascular disease, or any inflammatory, tumor, or infectious disease that could influence our results. All subjects were older than 18 years of age. Complete clinical information, including family and personal history, symptoms of HF, and pharmacological information, was obtained from each patient. Transthoracic echocardiogram and electrocardiogram were performed on all individuals. All patients underwent coronary-artery catheterization prior to surgery as recommended by the European Society of Cardiology guidelines [9]. Only 4 VCM and 4 ICM samples were used for mRNA-seq and 4 VCM and 3 ICM samples for miRNA-seq due to technical issues. The entire population of samples was used to validate the sequencing results.

### 2.2. Myocardial Tissue Collection

Human left ventricular transmural core biopsies were collected by experienced cardiothoracic surgeons at the Puerta del Mar Hospital University. Samples were obtained from 15 patients undergoing valve-replacement surgery or coronary-artery bypass grafting. After entering extracorporeal circulation and performing aortic clamping, the anterior descending coronary artery and the interventricular septum were located. A disposable 18 G/15 cm biopsy needle (Quick-core, Cook Medical, Bloomington, IN, USA) was used to extract the ventricular biopsy as described [28]. Samples were biopsied from the left anterolateral free wall between the anterior descending coronary artery and the marginal oblique, close to the apex. No complications were observed during this technique. Cardiac tissue samples were collected, frozen in liquid nitrogen, and stored at −80 °C.

### 2.3. RNA Isolation and qRT-PCR

All qRT-PCR experiments followed MIQE guidelines [29]. Total RNA was extracted and purified by using TRI Reagent (Sigma-Aldrich, St. Louis, MO, USA) according to the manufacturer’s instructions, followed by DNase treatment and purification using RNA clean and a concentrator-5 kit (Zymo Research, Irvine, CA, USA). RNA was quantified using a Qubit RNA High-Sensitivity Assay kit in the Qubit^®^ 2.0 Fluorometer (Life Technologies, Carlsbad, CA, USA). mRNA was reverse-transcribed employing a PrimeScript RT reagent Kit (Takara, Shiga, Japan) following the manufacturer’s instructions. Relative quantification of mRNA levels was executed using forward and reverse primers at 100 nM each (primer pairs shown in Table 1) and iTaq Universal SYBR Green Supermix (Bio-Rad, Hercules, CA, USA). The mRNA levels were normalized to GAPDH and ß-ACTIN. For miRNA expression analysis, 5 ng of RNA were reverse-transcribed using a miRCURY LNA RT Kit (Qiagen, Hilden, Germany) and amplified with hsa-miR-106b-3p (339306, Qiagen), hsa-miR-193b-5p (339306, Qiagen), hsa-miR-218-5p (339306, Qiagen), hsa-miR-487b-3p (339306, Qiagen), and hsa-miR-494-3p (339306, Qiagen) miRCURY LNA miRNA PCR primer set and a miRCURY LNA SYBR Green PCR Kit (Qiagen, Hilden, Germany) according to the manufacturer’s instruction. All qRT-PCRs were performed on a CFX96 Real-Time PCR system (Bio-Rad, Hercules, CA, USA). The miRNA expression was normalized against U6 snRNA (v2) and a 5S rRNA miRCURY LNA miRNA PCR primer set, and data were analyzed using the 2^−ΔΔCt^ algorithm.

### 2.4. RNA-Seq Analysis and Bioinformatics

The quality and integrity of the total RNA were controlled on the Agilent Technologies 2100 Bioanalyzer. Standard-specific mRNA sequencing (mRNA-seq) libraries were generated using the NEBNext Ultra II Directional RNA Library Prep Kit for Illumina using the NEBNext Poly(A) mRNA Magnetic Isolation Module (New England Biolabs, Ipswich, MA, USA), and single-end libraries were sequenced on an Illumina SE75 Platform with an output of ~70 M reads per sample. Standard miRNA libraries were generated using the NEXTFLEX small RNA-seq kit v3 (Perkin Elmer, Waltham, MA, USA), and single-end libraries were sequenced on an Illumina SE75 Platform with an output of ~20 M reads per sample.

For trimming and aligning raw data, fastq sequence reads were uploaded to the European version of the Galaxy platform [30]. Reads were trimmed with the Trim Galore software (Galaxy Version 0.6.7+galaxy0) and aligned to the built-in human reference genome, Dec. 2013 (GRCh38/hg38), with the RNA STAR Gapped-read mapper (Galaxy Version 2.7.10b+galaxy3) [31]. For gene-expression analyses, bam files were downloaded from the Galaxy server and further analyzed with the different RStudio packages downloaded from the Bioconductor website (http://bioconductor.org, accessed on 2 March 2023). Reads were assigned to genes by means of the “featureCounts” function of the “Rsubread” package, version 2.10.5. [32] and annotation files human release 43 (GRCh38.p13) (https://www.gencodegenes.org/human/release_43.html, accessed on 2 March 2023) and Chromosomal coordinates of Homo sapiens microRNAs (https://www.mirbase.org/ftp/CURRENT/genomes/hsa.gff3, accessed on 2 March 2023) for mRNA and miRNA analysis, respectively. Only the mapped reads were used to calculate gene expressions. The library size of each experimental point ranged from 58,431,140 to 76,443,844 sequences and from 7,530,361 to 27,506,550 sequences for mRNA and miRNA analysis, respectively. The differential gene-expression analyses were performed with package ‘DESeq2’ version 1.36.0 [https://doi.org/10.1186/s13059-014-0550-8, accessed on 2 March 2023]. All the gene comparisons with a *p* value < 0.05 and an abslog_2_ fold change (FC) > 0.5 were considered differentially expressed under the experimental conditions. For miRNA–target transcript interaction network analysis, first, we computed Pearson correlation coefficients for all miRNA–mRNA couples available in each group of samples (VCM and ICM). The next step consisted of intersecting the significant negative correlations with the predicted miRNA–mRNA potential interactions from DIANA-microT and ElMMo PITA databases [33,34]. In addition, in order to minimize the false-positive ratio, the information from experimentally validated miRNA–mRNA target pairs extracted from TarBase and miRTarbase databases was also implemented [35,36]. This experimental approach is similar to the one carried out by Vila-Casadesús et al. (2016) [37] previously. Functional enrichment analyses and Gene Set Enrichment Analysis (GSEA)-based [38] Kyoto Encyclopedia of Genes and Genomes (KEGG) and Gene Ontology (GO) analyses were conducted with the “clusterProfiler” package version 3.6.0. [39,40]. The gene sets with a *p*-value < 0.05 were considered overrepresented under the experimental conditions.

### 2.5. Luciferase Reporter Assay

Dual-luciferase reporter assay was performed as described previously [41].

DDX6, TTC39C, SEMA4A, and SGMS2 3’UTR fragments were PCR-amplified and cloned into the pMIR-REPORT vector. The 3T3 fibroblasts (ATCC) were cultured with DMEM (Gibco, Waltham, MA, USA) supplemented with 10% fetal bovine serum and 1% penicillin/streptomycin and seeded overnight at 50–60% confluence in 24-well plates. Then, the cells were co-transfected using Lipofectamine 3000 (Thermo Fisher Scientific, Waltham, MA, USA) with 100 ng of the luciferase vector carrying the 3’UTR fragment and 50 nM of the mirVana mimics has-miR-218-5p or has-miR-494-3p (4464066, Thermo Fisher Scientific, Waltham, MA, USA), along with 300 ng of pcLux vector control for internal normalization.

PCR-based site-directed mutagenesis was performed using the Bio-Rad iPROOF PCR kit. Primers, including MD218_DDX6_Fw (5′-GTCCCTCTTAAACCACAGAC-3′), MD218_DDX6_Rv (5′-GTCTGTGGTTTAAGAGGGAC-3′), MD218_SEMA4_Fw (5′-CTCAAGAGCAGAGAGA-3′), MD218_SEMA4_Rv (5′-TCTCTCTGCTCTTGAG-3′), MD494_SMGS2_Fw (5’-ACACTGCAGCTGCCAC-3’), and MD494_SMGS2_Rv (5’-GTGGCAGCTGCAGTGT-3’). The underlined nucleotides are referenced to nucleotides changed in site-directed mutagenesis and were used to introduce mutations into the sequence complementary to seed sequences of miR-218 and miR-494 within the 3′ UTRs of *DDX6*, *SEMA4*, and *SMGS2*, respectively. Each luciferase assay was carried out in triplicate and repeated in at least three distinct biological samples to obtain representative means.

Luciferase activity was measured 18 h after transfection by using the Pierce Gaussia Luciferase Flash Assay Kit (Thermo Fisher Scientific, Waltham, MA, USA) and normalized to pcLux vector control by using the Pierce Cypridina Luciferase Flash Assay Kit (Thermo Fisher Scientific, Waltham, MA, USA). In all cases, transfections were carried out in triplicate.

### 2.6. Statistical Analysis

Data are expressed as mean ± SEM, and n denotes the number of replicates for each experiment. Outliers were identified through the Rout method, using a Q = 1%. The normal distribution of each variable was verified with the Shapiro–Wilk test. Statistical differences (*p* < 0.05) between the experimental groups were assessed using a two-tailed, unpaired Student’s *t* test for Gaussian distributions. For non-Gaussian distributions, a Mann–Whitney non-parametric test was used [42]. All the statistical analyses were performed using GraphPad Prism 9.0 software (San Diego, CA, USA).

## 3. Results

### 3.1. Baseline Characteristics

The baseline characteristics of the 15 patients with DCM included in this study are shown in Table 2. The ICM cohort included six patients (mean age, 66.33 ± 7.84 years; all male patients) while the VCM group comprised nine patients (mean age, 63.67 ± 11.78 years; one female). There were no significant differences in the echocardiographic variables, such as left-ventricle ejection fraction, dilated left ventricular end-diastolic diameter, left ventricular end-systolic diameters, and left atrial dimension.

### 3.2. VCM and ICM Have Distinct mRNA and miRNA Expression Profiles

**Figure 1:**
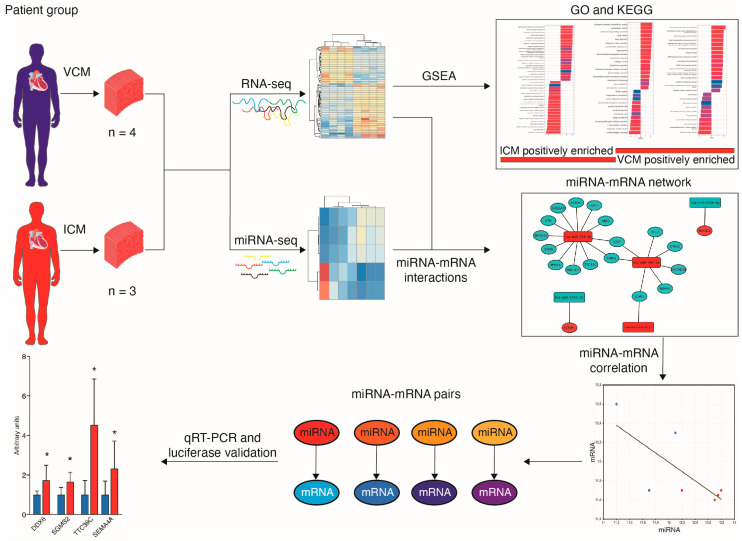
A flowchart of the study design * *p* < 0.05.

**Figure 2:**
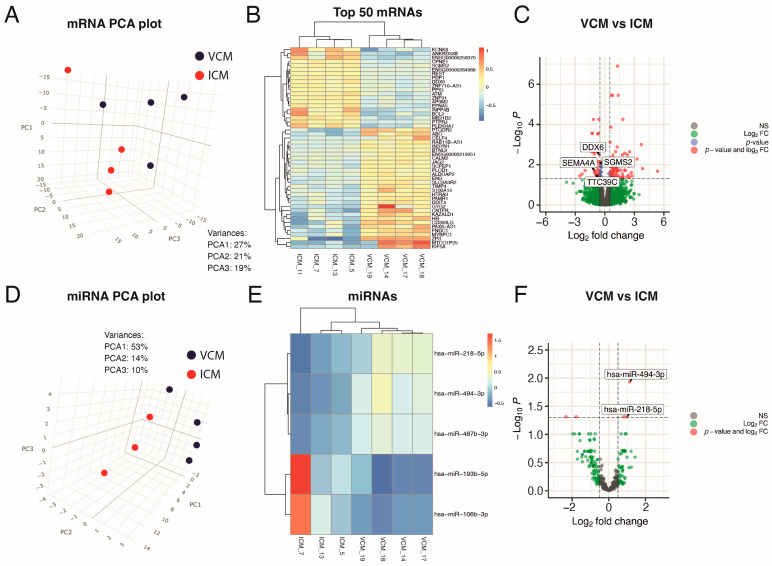
Exploratory analysis of paired miRNA and mRNA expression in heart samples. (**A**) 3D principal components analysis plot, based on correlation matrix, for mRNA expression in VCM (n = 4) and ICM (n = 4) tissue samples. (**B**) Heatmap of the top-50 most differentially expressed mRNAs sorted by absolute FC (all of them having FDR < 0.05). (**C**) Volcano plot of the mRNAs, highlighting in gray those not statistically significant, with FDR > 0.05 and absolute FC > 1.42 (abslog_2_FC > 0.5), in blue those with FDR < 0.05 but absolute FC > 1.42 (abslog_2_FC > 0.5), in green those with absolute FC > 1.42 (abslog_2_FC > 0.5) but FDR > 0.05, and in red those with FDR < 0.05 and absolute FC > 1.42 (abslog_2_FC > 0.5). (**D**) 3D principal components analysis plot, based on correlation matrix, for miRNA expression in VCM (n = 4) and ICM (n = 3) tissue samples. (**E**) Heatmap of the only 5 differentially expressed miRNAs sorted by absolute FC (all of them having FDR < 0.05). (**F**) Volcano plot of the miRNAs highlighting in gray those not statistically significant with FDR > 0.05 and absolute FC > 1.42 (abslog_2_FC > 0.5), in green those with absolute FC > 1.42 (abslog_2_FC > 0.5) but FDR > 0.05 and in red those with FDR < 0.05 and absolute FC > 1.42 (abslog_2_FC > 0.5).

To investigate the mRNA expression differences between DCM etiologies, we performed RNA-seq on left ventricular tissue from four VCM and three ICM patients (Figure 1). We used principal component analysis (PCA) to visualize sample clustering for the most variably expressed genes, and no outliers were observed. PCA indicated that the VCM samples are plainly different from the ICM ones based on mRNA profiling (Figure 2A).

The filtered RNA-seq read set identified 18,975 genes and 583 miRNAs. In total, 111 genes were differentially expressed between VCM and ICM, with an abslog_2_ fold change (FC) > 0.5 using a 5% false discovery rate (FDR) (Supplementary Table S1). Of those genes, 75 were upregulated and 36 were downregulated in VCM relative to ICM (Figure 2B,C).

We also analyzed the miRNA profile by miRNA-seq to investigate the differential expression between these distinct etiologies of DCM. No outliers were observed in the miRNA expression profile in a PCA. According to the PCA, the miRNA expression characterization of the VCM samples differed significantly from that of ICM biopsies (Figure 2D). After sequencing analysis, 634 miRNAs from all myocardial samples were identified. The most highly expressed miRNA in all the myocardial samples was miR-1-3p (Supplementary Table S2). Differential expression analysis identified five miRNAs to be differentially expressed between VCM and ICM (abslog_2_ FC > 0.5; FDR ≤ 0.05) (Supplementary Table S3). Whereas miR-218-5p, miR-487b-3p, and miR-494-3p were upregulated, miR-106b-3p and miR-193b-5p were downregulated in VCM compared to ICM, respectively (Figure 2E,F).

**Figure 3:**
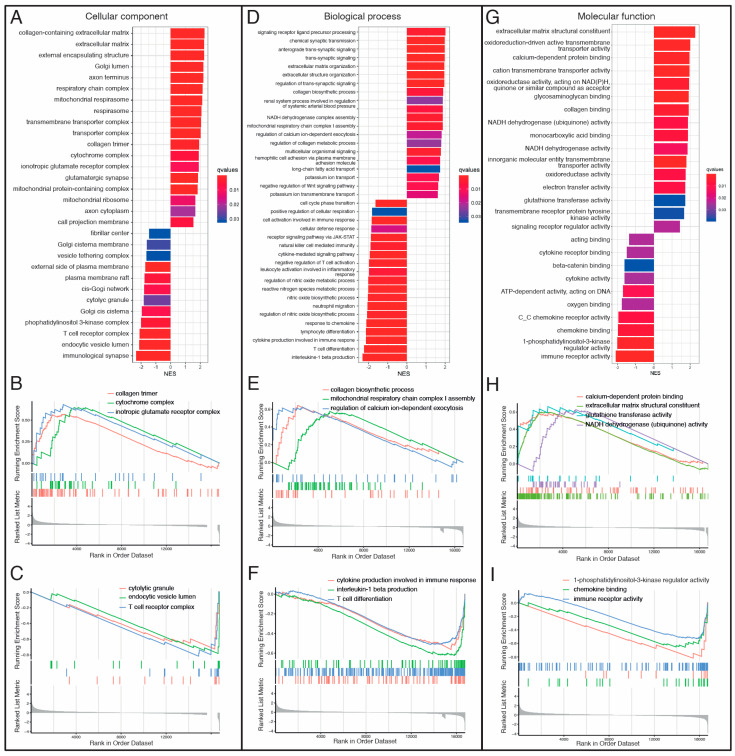
Gene Ontology (GO) Gene Set Enrichment Analysis (GSEA) between VCM and ICM groups. (**A**) Bar plot for cellular component (CC) analysis ranked by Normalized Enrichment Score (NES). (**B**) Gseaplot for “collagen trimer”, “cytochrome complex”, and “ionotropic glutamate receptor” upregulated GO terms showing the running score and preranked list of GSEA and its association of phenotype in CC analysis. (**C**) Gseaplot for “cytolytic granule”, “endocytic vesicle lumen”, and “T cell receptor complex” downregulated GO terms showing the running score and preranked list of GSEA and its association of phenotype in CC analysis. (**D**) Bar plot for biological process (BP) analysis ranked by Normalized Enrichment Score (NES). (**E**) Gseaplot for “collagen biosynthetic process”, “mitochondrial respiratory chain complex I assembly”, and “regulation of calcium ion-dependent exocytosis” upregulated GO terms showing the running score and preranked list of GSEA and its association of phenotype in BP analysis. (**F**) GSEA plot for “cytokine production involved in immune response”, “interleukin-1 beta production”, and “T cell differentiation” downregulated GO terms showing the running score and preranked list of GSEA and its association of phenotype in BP analysis. (**G**) Bar plot for molecular function (MF) analysis ranked by Normalized Enrichment Score (NES). (**H**) GSEA plot for “calcium-dependent protein binding”, “extracellular matrix structural constituent”, “glutathione transferase activity”, and “NADH dehydrogenase (ubiquinone) activity” upregulated GO terms showing the running score and preranked list of GSEA and its association of phenotype in MF analysis. (**I**) GSEA plot for “1-phosphatidylinositol-3-kinase regulator activity”, “chemokine binding”, and “immune receptor activity” downregulated GO terms showing the running score and preranked list of GSEA and its association of phenotype in MF analysis. NES: normalized enrichment scores.

**Figure 4:**
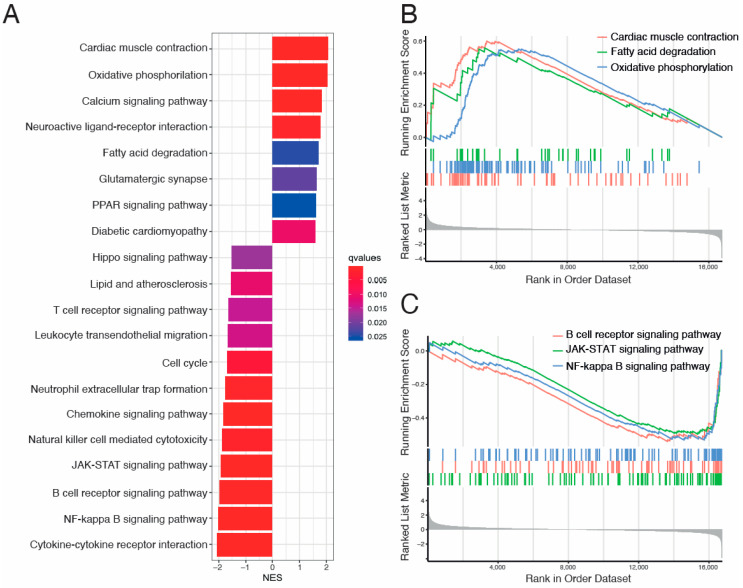
Kyoto Encyclopedia of Genes and Genomes (KEGG) Gene Set Enrichment Analysis (GSEA) between VCM and ICM groups. (**A**) Bar plot for KEGG analysis ranked by Normalized Enrichment Score (NES). (**B**) GSEA plot for “Cardiac muscle contraction”, “Fatty acid degradation”, and “Oxidative phosphorylation” upregulated KEGG terms showing the running score and preranked list of GSEA and its association of phenotype. (**C**) GSEA plot for “B cell receptor signaling pathway”, “JAK-STAT signaling pathway”, and “NF-kappa B signaling pathway” downregulated KEGG terms showing the running score and preranked list of GSEA and its association of phenotype. NES: normalized enrichment scores.

To identify pathways related to each DCM etiology, we performed pathway-enrichment analysis on the differentially expressed genes in our study. We ranked genes in order from increasing to decreasing abslog2-fold expression changes in VCM vs. ICM. Then, GO and KEGG pathway analyses based on GSEA were performed to evaluate key molecules and pathways contributing to each DCM etiology. The most enriched GO terms according to the gene ratio and categorized by the cellular component (CC), biological process (BP), and molecular function (MF) are shown in Figure 3A–I and Supplementary Tables S4–S6. GO analysis revealed extracellular matrix (ECM)-, mitochondria respiration- and immune-response-related pathways as the main terms involved in CC, BP, and MF (Figure 3A–I). In CC, collagen trimmer, cytochrome complex, and ionotropic glutamate receptor complex were among the most upregulated pathways in VCM compared to ICM (Figure 3B), whereas cytolytic granule, endocytic vesicle lumen, and T-cell receptor complex were among the most downregulated pathways (Figure 3C). In BP, collagen biosynthetic process, mitochondrial respiratory chain complex I assembly, and regulation of calcium ion-dependent exocytosis were among the most positively enriched pathways in VCM related to ICM (Figure 3D,E), whereas cytokine production involved in immune response, interleukine-1 (IL-1) beta production, and T-cell differentiation were among the most negatively enriched pathways (Figure 3D,F). Finally, calcium-dependent protein binding, ECM structural constituent, glutathione transferase activity, and NADH dehydrogenase (ubiquinone) activity were among the most positively enriched pathways in VCM related to ICM (Figure 3G,H), whereas 1-phosphatidylinositol-3-kinase regulator activity, chemokine binding, and immune-receptor activity were the most negatively enriched pathways in MF (Figure 3G,I). On the other hand, the results of the KEGG pathway analysis showed that cardiac muscle contraction, fatty acid degradation, and oxidative phosphorylation, among others, were positively enriched in VCM when compared to ICM (Figure 4A,B), whereas B cell receptor, JAK-STAT, and NF-kappa B signaling pathways, among others, were negatively regulated with VCM (Figure 4A,C).

### 3.3. miRNA–Target Transcript Interaction Network

**Figure 5:**
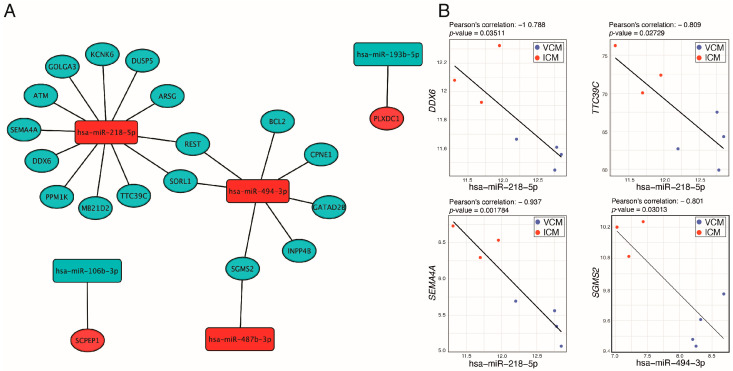
miRNA–miRNA interaction analysis. (**A**) Network of selected miRNA–mRNA interactions. (**B**) Negatively correlated miRNA–mRNA pairs predicted simultaneously, at least, in three used databases in our pipeline. Red and blue nodes mean upregulated and downregulated in VCM vs. ICM, respectively.

In order to identify miRNA–target pairs that could be playing an active role in the two etiologies of DCM, we focused on those miRNAs that were differentially expressed between VCM and ICM. We selected miRNA–target pairs from three prediction tools (DIANA-microT, ElMMo, and PITA,) and from databases that list experimentally validated miRNA–target pairs (TarBase and miRTarbase) (Table 3). We identified 22 miRNA–target transcript pairs. The resulting miRNA–target transcript interaction network consists of five miRNAs connected to 19 genes (Figure 5A). Of the 22 miRNA–transcript pairs, 13 miRNA–transcript interactions have been previously experimentally validated (Table 3). Afterward, we performed a Pearson correlation between the selected miRNAs and genes. This identified four negatively correlated miRNA–target transcript pairs, miR-218-5p-*DDX6*, miR-218-5p-*TTC39C*, miR-218-5p-*SEMA4A*, and miR-494-3p-*SGMS2* (R < −0.7 and *p*-value < 0.05) (Figure 5B), of which only miR-218-5p-DDX6 has been previously experimentally validated (Table 3).

**Figure 6:**
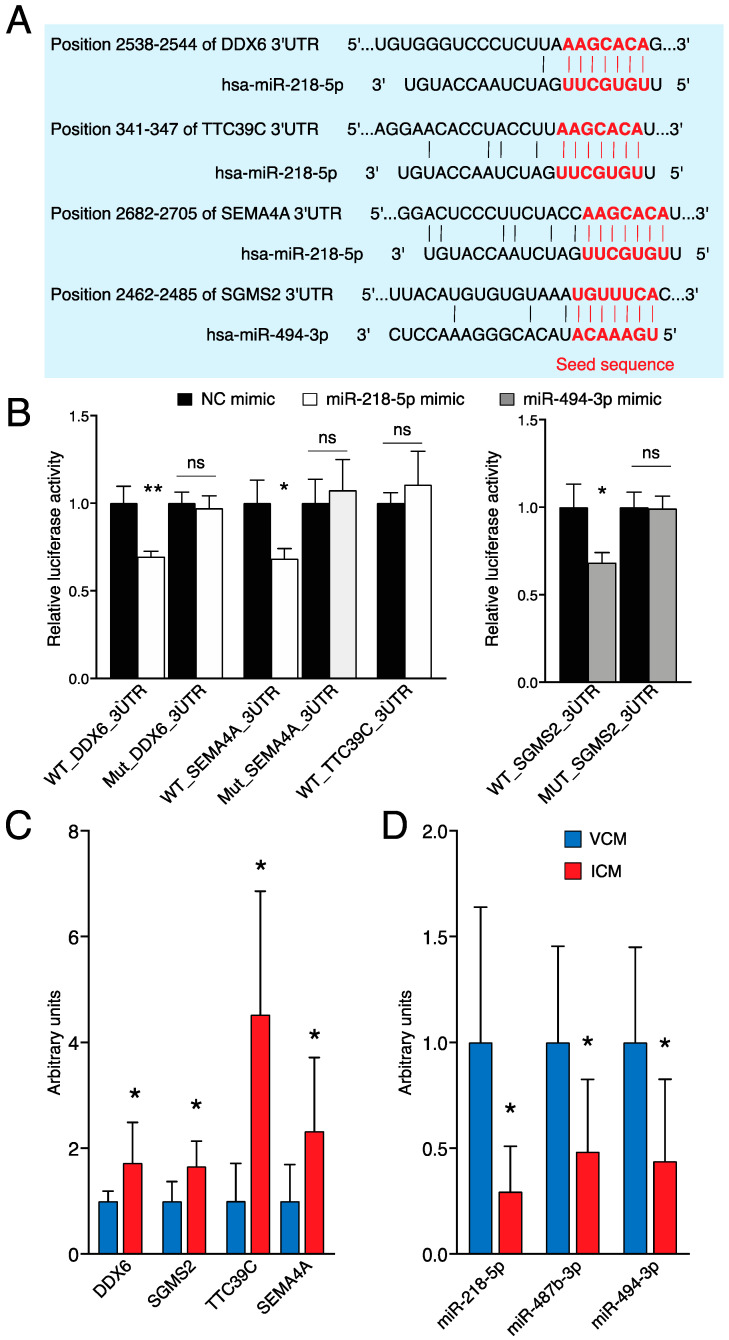
miR-218-5p targets DDX6 and SEMA4A and miR-494-3p targets *SGMS2*. (**A**) Predicted miR-218-5p binding sites in the 3’UTR of *DDX6*, *TTC39C*, and *SEMA4A*, and predicted miR-494-3p binding sites in the 3’UTR of *SGMS2*. The red nucleotides are referenced to seed sequence and sequence complementary to seed sequence of miRNA and 3’UTR, respectively. (**B**) Dual-luciferase activity assay in 3T3 cells co-transfected with the pMIR-REPORT miRNA expression reporter vector containing the wild-type (WT) or mutant (Mut) *DDX6*, *TTC39C*, or *SEMA4A* 3’UTR fragment with miR-218-5p mimic, and the pMIR-REPORT miRNA expression reporter vector containing the wild-type (WT) or mutant (Mut) *SGMS2* 3’UTR fragment with miR-494-3p mimic for 18 h (n = 3). (**C**) Expression levels of *DDX6*, *TTC39C*, *SEMA4A*, and *SGMS2* in VCM (n = 9) vs. ICM (n = 6) analyzed by qRT-PCR. (**D**) Expression levels of miR-218-5p, miR-487b-3p, and miR-494-3p in VCM (n = 9) vs. ICM (n = 6) analyzed by qRT-PCR. ** *p* < 0.01; * *p* < 0.05. ns: nonsignificant; NC: negative control.

To investigate the physical interaction of the potential miRNA–target transcript pairs, we performed a dual-luciferase reporter assay. The pMIR-REPORT containing the wild-type (WT) or mutant (Mut) *DDX6* 3’UTR, *TTC39C* 3’UTR, or *SEMA4A* 3’UTR was co-transfected with miR-218-5p mimic, and the pMIR-REPORT containing the wild-type (WT) or mutant (Mut) *SGMS2* 3’UTR was co-transfected with miR-494-3p mimic into 3T3 cells. Figure 6A shows the predicted miRNA binding site in the 3’UTR of its target gene.

Transfection of miR-218-5p mimic significantly reduced the luciferase activity in WT_*DDX6*_3’UTR and WT_*SEMA4A*_3’UTR, whereas the luciferase activity in the WT_*TTC39C*_3’UTR remained unaltered. Additionally, miR-494-3p significantly reduced the luciferase activity of SGMS2 (Figure 6B). Therefore, these results confirmed that *DDX6* and *SEMA4A* are direct targets of miR-218-5p, and *SGMS2* is a direct target of miR-494-3p.

### 3.4. Validation of Differentially Expressed Genes and miRNAs by qRT-PCR

To validate the sequencing data for certain differentially expressed genes, miRNAs were verified in the myocardial tissue of nine VCM and six ICM patients by qRT-PCR. *ß-ACTIN* and *GAPDH* were used as an internal control for mRNA qRT-PCRs, whereas U6 and 5s small nuclear RNAs were used for miRNAs. As presented in Figure 6D, significant differences were observed in the expression of the four deregulated genes between the VCM and ICM groups. *DDX6*, *SGMS2*, *TTC39C*, and *SEMA4A* were upregulated in ICM heart samples compared to VCM (Figure 6D). On the other hand, only the expressions of miR-218-5p, miR-487b-3p, and miR-494-3p, downregulated in ICM samples compared to VCM, were comparable with the sequencing data (Figure 6E).

## 4. Discussion

DCM is commonly regarded as a final pathway resulting from various underlying causes, which significantly influences both prognosis and outcomes [43]. However, there remains a significant gap in knowledge regarding etiologically based biological pathways associated with DCM. Here, we propose an approach to better understand molecular and cellular processes of distinct DCM etiologies through transcriptome analysis of mRNA and miRNA. Although several studies have analyzed and compared the transcriptome of distinct DCM etiologies against healthy controls [19,23,24], to date, studies comparing the mRNA and miRNA transcriptome of different etiologies with each other remain poor. Using RNA-seq, we identified genes that are differentially expressed between VCM and ICM myocardial biopsies. Pathway analysis with GSEA revealed ECM- and mitochondrial respiration-related pathways as the most common positively enriched biological routes for the GO terms CC, BP, and MF in VCM compared to ICM. In contrast, immune-activation-related pathways were among the most negatively enriched in VCM vs. ICM.

In DCM, the deposition of structural ECM proteins varies based on the mechanical or ischemic trigger. Thus, several studies suggest that, in contrast to the marked deposition of structural ECM due to mechanical alterations (i.e., pressure overload) or injuries (myocardial infarction), volume overload is less profibrotic [44,45]. VCM develops a compensatory response to maintain ventricular shape and size. However, fibrosis in VCM is associated with the loss of interstitial collagen, a prominent ECM degradation [46,47,48,49]. Although the collagen gene expression increases, there is an imbalance in the matrix metalloproteases (MMP) and tissue inhibitor metalloproteases (TIMPs) that correlates with unaltered collagen protein levels [49]. In this sense, Irqsusi et al. demonstrated that MMPs were expressed during mitral valve regurgitation, a model of volume overload, in a dynamic destruction process supported by TIMPs, the expression of which increased with the severity of the mitral regurgitation [50]. Accordingly, our results showed the upregulation of collagen and MMP family genes. The fact that both collagen synthesis and ECM degradation pathways are activated during ventricular remodeling in VCM may explain the enrichment of the ECM-related pathway compared to ICM, even being less fibrotic.

ICM fibrosis is the consequence of a repair process secondary to significant cardiomyocyte death that stimulates the inflammation cascade and the activation of reparative myofibroblasts to protect myocardium integrity. Cardiomyocyte death rapidly activates innate immune pathways that trigger cytokine, chemokine, and adhesion molecule expression, initiating the inflammatory phase and leading to the infiltration of the infarct by leukocytes and T cells [51]. Accordingly, our results showed positive enrichment of immune-response-related pathways in ICM compared to VCM. In addition, biopsy samples collected from the remote zone of ICM hearts are subject to diffuse reactive fibrosis that differs from the reparative fibrosis that occurs in the infarcted zone [52] GSEA analysis also revealed mitochondrial respiration-related pathways as more enriched in VCM compared to ICM, suggesting a more severe mitochondrial dysfunction in the ICM cohort. It is well known that mitochondrial dysfunction contributes to the transition from the normal heart to end-stage-HF, which is the common pathway of DCM, including VCM and ICM [53,54,55]. Therefore, the enrichment of pathways related to mitochondrial respiration in VCM may indicate a more severe mitochondrial dysfunction in ICM than in VCM. It has been suggested that mitochondrial dysfunction may represent only a late phenomenon in the disease process. Therefore, these results may indicate that ICM hearts go into failure faster than VCM. Consistently, rats with infarcted hearts displayed clear impairment and mitochondrial dysfunction at baseline, whereas a model of compensated volume overload showed the absence of major mitochondrial and contractile dysfunction [56,57]. Mitochondrial ion channels allow the fine-tuning of its membrane potential, ROS production, and function of the respiratory chain complexes and are extensively studied in terms of protecting the heart or cardiac cells from ischemia–reperfusion injury [58]. This is consistent with the negative enrichment of mitochondrial respiratory-related pathways, such as respiratory complex I and the cytochrome complex in ICM related to VCM. On the other hand, the ICM cohort presented a downregulation of calcium ion-dependent exocytosis and calcium-dependent protein-binding pathways, which is also related to mitochondrial dysfunction and ischemic heart disease. Ischemia–reperfusion induces a large influx of calcium into the cell and the mitochondrial outer compartment. The subsequent opening of the membrane permeability transition pore in the inner mitochondrial membrane and the resulting calcium overload induces the homeostasis of cardiomyocytes and activates the mitochondrial pathway of apoptosis [59]. Hence, our results support the idea that mitochondria are strongly recommended as a therapeutic target for cardiac ischemia–reperfusion injury [59,60]. It is well-recognized that cardiac ischemia–reperfusion injury following myocardial infarction, is a complex process involving multiple cellular and molecular pathways. Targeting mitochondria offers a promising approach due to their central role in energy production, oxidative stress, and cell-death pathways. Mitochondria-targeted therapies, including antioxidants, mitochondrial uncouplers, and agents that modulate mitochondrial permeability transition, have shown potential in preclinical studies. However, translating these findings into effective clinical therapies remains challenging due to issues such as off-target effects, limited tissue specificity, and inadequate delivery methods [60]. Hence, our findings underscore the importance of mitochondria as therapeutic targets in the management of ICM.

Finally, KEGG enrichment analysis showed cardiac muscle contraction and fatty acid degradation among the most positively enriched pathways in VCM compared to ICM. An enrichment of the cardiac muscle contraction pathway in VCM could be explained by the fact that volume overload is characterized by an eccentric (lengthening) muscle contraction as a consequence of the mechanical stress [61]. Fatty acids are the main energy substrate of the heart via ß-oxidation to produce ATP in mitochondria [61]. Therefore, fatty acid degradation pathway enrichment could be a secondary effect of the less dysfunctional state of mitochondria in VCM. In this sense, we also observed a positive enrichment of the PPAR signaling pathway, involved in fatty acid metabolism and mitochondrial function in the heart [62].

miRNA-seq analysis resulted in five miRNAs deregulated. miR-218-5p, miR-487b-3p, and miR-494-3p were upregulated, whereas miR-106b-3p and miR-193b-5p appeared downregulated in VCM vs. ICM. Among the 22 predicted miRNA–target transcript pairs, only four were negatively correlated, miR-218-5p-*DDX6*, miR-218-5p-*TTC39C*, miR-218-5p-*SEMA4A*, and miR-494-3p-*SGMS2*, and only three (miR-218-5p-*DDX6*, miR-218-5p-*SEMA4A*, and miR-494-3p-*SGMS2*) displayed physical interaction. DDX6 is an RNA helicase with roles in cellular stress and hypoxia [63], processing (P)-body homeostasis regulating stem-cell plasticity [64], and the miRNA pathway [65,66]. In this regard, our analysis shows that the *DDX6* gene is involved in P-body formation in CC, the regulation of cellular macromolecule biosynthetic processes in BP, and is associated with double-stranded RNA binding in MF. Although little is known about its role in the heart, DDX6 has been associated with abnormal heart formation and described as modulating bone morphogenic protein (BMP) signaling [67]. The BMP subfamily belongs to the transforming growth factor beta (TGF-β) superfamily, which is well-documented to be upregulated in animal models of myocardial infarction [68]. In addition, BMP subfamily members have been suggested to exert both pro- and anti-inflammatory actions and may regulate fibrosis in myocardial infarction. Curiously, miR-218-5p, a potential miRNA targeting *DDX6*, has been also reported to mediate myocardial fibrosis and inflammation after myocardial infarction [69,70,71,72], suggesting therefore that the so far unknown *DDX6*/miR-218-5p axis could play a regulatory role during fibrosis and inflammation in the infarcted heart. Regarding the SEMA4A, which is involved in cell activation, immune response, T-cell differentiation, and lymphocyte differentiation according to BP, an in vivo cardiac ischemia–reperfusion mouse model showed *SEMA4A* to be highly expressed in macrophages recruited at the injured area, and it was suggested to exert a role activating angiogenesis and inflammation [73]. Accordingly, our results showed higher expression of *SEMA4A* in ICM than in VCM. Interestingly, miR-218-5p has been associated with inflammation in a myocardial ischemia–reperfusion injury model [71], suggesting, therefore, the *SEMA4A*/miR-218-5p axis as a potential target for regulating cardiac inflammation. It is well-known that the inflammatory response following an ischemic event can lead to adverse remodeling, cardiomyocyte injury, and matrix degradation, ultimately contributing to the development of HF. Targeting specific inflammatory pathways can potentially reduce infarct size, promote repair, and prevent adverse remodeling, offering a promising approach to improve outcomes in patients with ICM [74]. This positions inflammation as a therapeutic target for ICM in the context of diversifying therapies against ICM vs. VCM.

So far, nothing is known about the role of TTC39C in the heart. In fact, *TTC39C* is a little-studied gene. A Pubmed search only resulted in 11 results, and its function is unknown. To date, it has been only related to immunodeficiency [75]. Therefore, further studies are needed to unveil the role of this gene in heart disease. Finally, Sphingomyelin synthase 2 (*SGMS2*, also *SMS2*) was upregulated in ICM hearts compared to VCM. Concordantly, *SGMS2* was found upregulated in cardiomyocytes in response to hypoxia in vitro [76]. This study concluded that downregulation of *SGMS2* may protect cardiomyocytes against hypoxia-induced apoptosis and oxidative stress by enhancing the expression of nuclear factor erythroid 2-related factor 2 (*Nrf2*), an inhibitor of apoptosis [77]. Interestingly, two studies have reported miR-494-3p, which constitutes a miRNA–target transcript pair together *SGMS2*, to play a protective role in myocardial ischemia–reperfusion injury, repressing inflammation and apoptosis [78,79]. In addition, SGMS2 has been also described to attenuate inflammatory injury after cerebral ischemia–reperfusion in mice [80]. This indicates that the *SGMS2*/miR-494-3p axis may be a novel potential target against hypoxia-induced inflammation and/or apoptosis in cardiomyocytes. Treating cardiomyocyte apoptosis in ICM vs. VCM is important because it directly addresses the underlying mechanisms driving HF in each condition. In ICM, cardiomyocyte apoptosis occurs as a result of ischemic injury, leading to a loss of functional heart muscle tissue. This process significantly contributes to the progression of HF by reducing the heart’s ability to pump effectively. In contrast, in VCM, cardiomyocyte apoptosis may be less prominent and may occur due to factors related to valvular dysfunction rather than ischemia [81]. Therefore, targeting cardiomyocyte apoptosis in ICM could be essential to halt the progression of HF and improve outcomes for affected individuals.

To date, most studies have tested differentially expressed genes between ischemic and non-ischemic cardiomyopathy relative to a normal heart [2,23,82,83,84], offering novel insight into unique disease-specific gene expression that exists between different etiologies. However, our approach goes beyond this by directly comparing the transcriptomic profiles of two distinct etiologies of DCM: VCM and ICM. This direct comparison allows us to identify unique gene-expression signatures associated with each etiology, as well as common pathways dysregulated in DCM as a whole. Understanding these differences is crucial for developing personalized treatments tailored to each etiology, potentially improving patient outcomes. Our study offers insights into the molecular heterogeneity of DCM and paves the way for more precise therapeutic interventions.

In conclusion, we found distinct gene-expression profiles in VCM and ICM heart tissues, indicating that distinct mechanisms are involved in the progression of the diseases towards end-stage HF. In addition, we found five differentially expressed miRNAs and identified four previously undescribed miRNA–target transcript pairs that may open new avenues for research into the molecular pathogenesis of DCM.

Despite the valuable insights provided by our study, it is crucial to acknowledge limitations. The small number of differentially expressed miRNAs observed between VCM and ICM is notable. This limitation is largely due to the difficulty in accessing human myocardial tissue samples. Additionally, the inherent variability in human tissue samples complicates analysis and may contribute to the modest number of differentially expressed miRNAs identified. Nevertheless, these findings offer valuable insights into the molecular differences between VCM and ICM, highlighting the potential role of miRNAs in DCM etiologies. Future research with larger sample sizes and comprehensive analyses will be essential to address these limitations and advance our understanding of DCM pathogenesis.

## Figures and Tables

**Table 1:** Quantitative real-time polymerase chain reaction primer pair sequence.

Gene	Forward	Reverse
*DDX6*	5′-AATACTGAACTATGGACCTATGAGCA-3′	5′-TTGCAGGGCTCACACTAGG-3′
*SEMA4A*	5′-TGGGGACTACTCTGCCTACTACA-3′	5′-GGGTTACTCTGCTCCATGTCA-3′
*SGMS2*	5′-AGCACGTGCACAGCTTCA-3′	5′-GTCCACGGGTGAAACAGC-3′
*TTC39C*	5′-TCTGGACAAGTACAATGCTGAGA-3′	5′-TAAGCTTCGCTGCACAGGT-3′
*GAPDH*	5′-AGCCACATCGCTCAGACAC-3′	5′-AATACGACCAAATCCGTTGACT-3′
*ß-ACTIN*	5′-TGTGGCATCCACGAAACTACC-3′	5′-CTCAGGAGGAGCAATGATCTTGAT-3′

**Table 2:** Baseline patient characteristics of the ICM and VCM groups. ICM: ischemic cardiomyopathy; VCM: volume overload cardiomyopathy; LVEF: Left-ventricle ejection fraction; LVEDD: left-ventricle end-diastolic diameter; LVESD: Left-ventricle end-systolic diameter; LA: left-atrium diameter.

Variable	ICM (N = 6)	VCM (N = 9)	*p*-Value
Age (years), means ± SD	66.33 ± 7.84	63.67 ± 11.78	0.637
Sex (male, %)	100	88.89	0.699
LVEF (%)	38 ± 15.87	48.13 ± 10.88	0.164
LVEDD (mm)	60.33 ± 2.16	60 ± 5.15	0.885
LVESD (mm)	44 ± 2.16	44.13± 7.62	0.969
LA (mm)	47.50 ± 2.65	52 ± 5.61	0.092
High blood pressure (%)	66.67	88.89	0.574
Dyslipidemia (%)	66.67	55.56	0.975
Diabetes Mellitus (%)	50	66.67	0.519
ACEI/ARAII (y/n, %)	83.33	77.77	0.792
Diuretics (y/n, %)	83.33	88.89	0.757
Calcium antagonist (y/n, %)	16.67	22.22	0.792
Statins (y/n, %)	83.33	77.78	0.792
Ezetrol (%)	16.67	22.22	0.792
Metformin (%)	33.33	33.33	>0.999
Metformin/IGP4 (%)	16.67	22.22	0.792
Insulin (%)	16.67	22.22	0.792
Aspirin (y/n, %)	100	22.22	0.003
Beta-blocker (%)	83.33	77.78	0.792

**Table 3:** miRNA–target pairs from three prediction tools, -DIANA-microT, ElMMo and PITA-, and from databases that list experimentally validated miRNA–target pairs (TarBase and miRTarbase).

Database	Mature Mirna acc	Mature Mirna ID	Target Symbol	Target Entrez	Target Ensembl	Type	Pubmed ID	Score
mirtarbase	MIMAT0000275	hsa-miR-218-5p	ATM	472	ENSG00000149311	validated	23212916	
mirtarbase	MIMAT0002816	hsa-miR-494-3p	BCL2	596	ENSG00000171791	validated	24960059	
mirtarbase	MIMAT0000275	hsa-miR-218-5p	DDX6	1656	ENSG00000110367	validated	23212916	
mirtarbase	MIMAT0000275	hsa-miR-218-5p	GOLGA3	2802	ENSG00000090615	validated	23212916	
mirtarbase	MIMAT0002816	hsa-miR-494-3p	REST	5978	ENSG00000084093	validated	23446348	
mirtarbase	MIMAT0000275	hsa-miR-218-5p	KCNK6	9424	ENSG00000099337	validated	23313552	
mirtarbase	MIMAT0000275	hsa-miR-218-5p	ARSG	22901	ENSG00000141337	validated	23622248	
mirtarbase	MIMAT0000275	hsa-miR-218-5p	MB21D2	151963	ENSG00000180611	validated	23212916	
tarbase	MIMAT0000275	hsa-miR-218-5p	REST	5978	ENSG00000084093	validated	20371350	
tarbase	MIMAT0003180	hsa-miR-487b-3p	SGMS2	166929	ENSG00000164023	validated	24038734	
tarbase	MIMAT0002816	hsa-miR-494-3p	CPNE1	8904	ENSG00000214078	validated	25653011	
tarbase	MIMAT0004672	hsa-miR-106b-3p	SCPEP1	59342	ENSG00000121064	validated	22291592	
diana_microt	MIMAT0000275	hsa-miR-218-5p	DUSP5	1847	ENSG00000138166	predicted		0.992
diana_microt	MIMAT0002816	hsa-miR-494-3p	SGMS2	166929	ENSG00000164023	predicted		0.978
diana_microt	MIMAT0002816	hsa-miR-494-3p	INPP4B	8821	ENSG00000109452	predicted		0.889
diana_microt	MIMAT0002816	hsa-miR-494-3p	GATAD2B	57459	ENSG00000143614	predicted		0.85
elmmo	MIMAT0000275	hsa-miR-218-5p	TTC39C	125488	ENSG00000168234	predicted		0.712
elmmo	MIMAT0000275	hsa-miR-218-5p	SEMA4A	64218	ENSG00000196189	predicted		0.63
elmmo	MIMAT0000275	hsa-miR-218-5p	SORL1	6653	ENSG00000137642	predicted		0.533
elmmo	MIMAT0002816	hsa-miR-494-3p	GATAD2B	57459	ENSG00000143614	predicted		0.505
elmmo	MIMAT0002816	hsa-miR-494-3p	SORL1	6653	ENSG00000137642	predicted		0.504
diana_microt	MIMAT0004767	hsa-miR-193b-5p	PLXDC1	57125	ENSG00000161381	predicted		0.831

## Data Availability

RNA-seq data generated in this manuscript have been deposited in GEO (Gene Expression Omnibus) of NCBI under accession code GSE243406 study.

## Supplementary Materials

The following supporting information can be downloaded at https://www.mdpi.com/article/10.3390/biom14050524/s1: Table S1: differentially expressed genes; Table S2: miRNA raw counts table; Table S3: differentially expressed miRNAs; Table S4: top 30 enriched GO terms categorized by the CC. Table S5: top 40 enriched GO terms categorized by the BP; Table S6: top 26 enriched GO terms categorized by the MF.
