# Supplementary material for: Unraveling the Etiology of Dilated Cardiomyopathy through Differential miRNA–mRNA Interactome
Source: Biomolecules. 2024 Apr 27;14(5):524. doi: 10.3390/biom14050524 (PMC11117812; doi:10.3390/biom14050524)
Supplement: Supplementary file 1 [file biomolecules-14-00524-s001.zip › biomolecules-2969409-supplementary.pdf]

Supplementary Table S1: differentially expressed genes

baseMean	log2FoldChange	lfcSE	stat	P value	P adj	Gene symbol	ENTREZID
103.3716	5.35854	1.363896	3.928849	8.54E-05	0.020873	SNAP25	6616
33.9948	3.837305	0.948362	4.046243	5.2E-05	0.015548	SYT1	6857
86.96979	3.710857	0.752974	4.928266	8.3E-07	0.000985	MTCO1P25	107075167
60.73746	3.274763	0.739737	4.426931	9.56E-06	0.00521	KIF5A	3798
514.7249	2.774374	0.749374	3.702254	0.000214	0.036005	HMGCS2	3158
16.71563	2.700608	0.699793	3.859154	0.000114	0.024239	SULT4A1	25830
31.97241	2.56912	0.611245	4.203091	2.63E-05	0.010361	CELF4	56853
77.29427	2.51401	0.537682	4.675641	2.93E-06	0.002263	GYG2	8908
25.48023	2.511295	0.693126	3.623144	0.000291	0.043353	DLGAP3	58512
15.0895	2.464574	0.594882	4.14296	3.43E-05	0.011767	TTR	7276
14.84678	2.421989	0.646515	3.746225	0.00018	0.032434	SNX31	169166
23.24875	2.290447	0.501649	4.565831	4.98E-06	0.00318	ENSG00000219951	
12.87241	2.227822	0.607712	3.66592	0.000246	0.039719	MAST1	22983
22.87867	2.082218	0.525712	3.960755	7.47E-05	0.01915	TBX1	6899
348.6463	2.032248	0.438467	4.634899	3.57E-06	0.002647	ZP3	7784
178.8616	2.017945	0.506032	3.987785	6.67E-05	0.018449	SLN	6588
104.2377	1.844862	0.504272	3.658469	0.000254	0.040539	CHRDL2	25884
27.40134	1.836501	0.500773	3.667336	0.000245	0.039719	PLCH1	23007
122.9845	1.828365	0.326365	5.602208	2.12E-08	5.6E-05	ABO	28
152.4626	1.790552	0.391844	4.569551	4.89E-06	0.00318	CNTFR	1271
26.67171	1.786303	0.469495	3.804734	0.000142	0.028291	ELMOD1	55531
271.9282	1.631335	0.447927	3.641966	0.000271	0.042497	CBLN1	869
443.9272	1.625474	0.409103	3.973266	7.09E-05	0.018803	POMZP3	22932
249.3629	1.38769	0.349292	3.972862	7.1E-05	0.018803	SRRM3	222183
243.2669	1.35853	0.304791	4.457257	8.3E-06	0.004808	HR	55806
478.8984	1.351987	0.217305	6.221595	4.92E-10	3.53E-06	PTGDR2	11251
39.4704	1.345293	0.361061	3.725943	0.000195	0.034222	LINC00968	100507632
62.21979	1.286092	0.35425	3.630463	0.000283	0.043335	GALNT8	26290
729.9243	1.284844	0.187198	6.863549	6.72E-12	1.24E-07	FNDC1	84624
181.954	1.280162	0.299754	4.270708	1.95E-05	0.008599	MYBPC1	4604
135.213	1.269714	0.323492	3.92503	8.67E-05	0.020873	WNT5B	81029
2440.949	1.251981	0.309693	4.042653	5.28E-05	0.015548	CES1	1066
1127.406	1.247156	0.257389	4.845421	1.26E-06	0.001301	CD300LG	146894
145.6204	1.224895	0.305863	4.00472	6.21E-05	0.017612	PNCK	139728
290.4635	1.192825	0.307229	3.88253	0.000103	0.022784	SDK1	221935
579.3032	1.173555	0.272409	4.308063	1.65E-05	0.008033	PAX8-AS1	654433
282.752	1.127178	0.298179	3.780212	0.000157	0.029635	PLP1	5354
68.43529	1.124438	0.263915	4.260608	2.04E-05	0.008787	ALDOAP2	228
9526.826	1.100775	0.271034	4.061384	4.88E-05	0.014822	PDLIM3	27295
159.0515	1.089789	0.261645	4.165139	3.11E-05	0.011301	S100A14	57402
515.4124	1.087234	0.231416	4.698179	2.62E-06	0.002211	KAZALD1	81621
559.3808	1.025175	0.283343	3.618138	0.000297	0.043648	TSKU	25987

Supplementary Table S1 (continued)

baseMean	log2FoldChange	lfcSE	stat	P value	P adj	Gene symbol	ENTREZID
363.7425	1.023621	0.277085	3.694255	0.000221	0.036494	LRRN4CL	221091
288.3557	0.942637	0.236898	3.979078	6.92E-05	0.018803	SYT13	57586
109.8005	0.917669	0.213297	4.302304	1.69E-05	0.008033	RAB11B-AS1	100507567
1447.357	0.903622	0.22559	4.005598	6.19E-05	0.017612	CRISPLD2	83716
1093.742	0.882909	0.216842	4.071662	4.67E-05	0.014419	NUDT8	254552
231.6893	0.851143	0.216832	3.925355	8.66E-05	0.020873	FABP5P9	642956
435.5182	0.838323	0.233572	3.589148	0.000332	0.047666	BHMT2	23743
1399.806	0.835675	0.21704	3.850328	0.000118	0.024844	PDE2A	5138
1287.517	0.829954	0.227096	3.654647	0.000258	0.040796	PLTP	5360
534.0874	0.82385	0.181072	4.549858	5.37E-06	0.003316	TIMP4	7079
551.4224	0.817122	0.209826	3.894282	9.85E-05	0.022536	FABP5P11	266699
1053.513	0.815254	0.195935	4.160841	3.17E-05	0.011301	PLXDC1	57125
4384.867	0.813414	0.214492	3.792286	0.000149	0.029121	FABP5	2171
797.6324	0.78325	0.127307	6.152459	7.63E-10	3.53E-06	PAMR1	25891
3232.742	0.760725	0.203358	3.740816	0.000183	0.032688	TSC22D3	1831
1602.826	0.741777	0.178089	4.165212	3.11E-05	0.011301	DDIT4	54541
196.2882	0.739654	0.188755	3.918586	8.91E-05	0.020896	RPL12P8	645161
435.4516	0.706236	0.17292	4.084168	4.42E-05	0.013896	PID1	55022
188.9824	0.703189	0.193931	3.62597	0.000288	0.043353	RPL12P12	85824
172.1906	0.678633	0.179464	3.78145	0.000156	0.029635	NMNAT2	23057
1229.824	0.667986	0.183592	3.638437	0.000274	0.042721	PCSK6	5046
9295.354	0.666469	0.183889	3.624304	0.00029	0.043353	TNXB	7148
393.1214	0.664973	0.169633	3.920071	8.85E-05	0.020896	HIGD1B	51751
8758.007	0.663834	0.107194	6.192819	5.91E-10	3.53E-06	ENG	2022
25666.23	0.654682	0.159109	4.114681	3.88E-05	0.012832	TIMP3	7078
4131.618	0.620828	0.145177	4.276363	1.9E-05	0.008588	HTRA3	94031
1732.045	0.611131	0.112666	5.424274	5.82E-08	0.000135	JAG2	3714
2493.125	0.564403	0.145112	3.88942	0.0001	0.022712	ITIH5	80760
11281.08	0.560132	0.131909	4.246353	2.17E-05	0.008949	SLC9A3R2	9351
795.0078	0.549035	0.131603	4.171918	3.02E-05	0.011301	SIGIRR	59307
2378.357	0.540611	0.120821	4.474482	7.66E-06	0.004579	SCPEP1	59342
3683.29	0.540212	0.107711	5.015378	5.29E-07	0.000755	INSYN1	388135
1546.82	0.51172	0.135884	3.765867	0.000166	0.030457	ITPKB	3707
1626.586	-0.50063	0.115177	-4.34662	1.38E-05	0.006925	ZNF710-AS1	109729181
594.9081	-0.50778	0.105718	-4.80319	1.56E-06	0.001523	REST	5978
1053.006	-0.51327	0.135881	-3.77739	0.000158	0.029669	GATAD2B	57459
492.0889	-0.52163	0.107625	-4.84671	1.26E-06	0.001301	ENSG00000284968	
937.4188	-0.54001	0.12711	-4.24839	2.15E-05	0.008949	SGMS2	166929
5144.709	-0.54007	0.109694	-4.92347	8.5E-07	0.000985	PDP1	54704
3741.083	-0.5465	0.119406	-4.57682	4.72E-06	0.00318	DDX6	1656
967.0277	-0.58452	0.15342	-3.80992	0.000139	0.028005	SRRM1	10250
792.7498	-0.60764	0.107997	-5.62647	1.84E-08	5.6E-05	AP3M2	10947

Supplementary Table S1 (continued)

baseMean	log2FoldChange	lfcSE	stat	P value	P adj	Gene symbol	ENTREZID
224.9062	-0.6465	0.173855	-3.71863	0.0002	0.034375	HERC5	51191
750.8128	-0.64772	0.123615	-5.23984	1.61E-07	0.000271	CPNE1	8904
229.1427	-0.66726	0.181759	-3.6711	0.000242	0.039612	OAS1	4938
981.0647	-0.67392	0.136575	-4.93443	8.04E-07	0.000985	ZNF91	7644
494.5421	-0.69485	0.173609	-4.00235	6.27E-05	0.017612	LIG4	3981
1877.34	-0.7264	0.187327	-3.87773	0.000105	0.022784	GOLGA3	2802
240.3047	-0.73026	0.196072	-3.72447	0.000196	0.034222	ARSG	22901
799.4427	-0.73057	0.140303	-5.20708	1.92E-07	0.000296	EFNA5	1946
911.7908	-0.73248	0.168461	-4.34807	1.37E-05	0.006925	ATM	472
922.8021	-0.75298	0.183522	-4.10292	4.08E-05	0.013265	SORL1	6653
393.4003	-0.79648	0.168447	-4.72838	2.26E-06	0.002097	PTPRJ	5795
12694.58	-0.85002	0.228376	-3.72204	0.000198	0.034231	PPM1K	152926
1484.824	-0.85618	0.237822	-3.60009	0.000318	0.046062	GABRA4	2557
119.5643	-0.92777	0.245333	-3.7817	0.000156	0.029635	TTC39C	125488
206.7986	-1.03228	0.266253	-3.87707	0.000106	0.022784	XRRA1	143570
602.8123	-1.0365	0.26943	-3.84702	0.00012	0.024898	DUSP5	1847
606.1124	-1.04682	0.222698	-4.70064	2.59E-06	0.002211	PLEKHA7	144100
64.92311	-1.04794	0.264951	-3.95523	7.65E-05	0.01915	SEMA4A	64218
2724.318	-1.06424	0.230888	-4.60934	4.04E-06	0.00288	INPP4B	8821
1113.706	-1.11171	0.250507	-4.43785	9.09E-06	0.005103	BCL2	596
215.349	-1.1523	0.204066	-5.64668	1.64E-08	5.6E-05	MB21D2	151963
462.0421	-1.26546	0.240751	-5.25628	1.47E-07	0.000271	ENSG00000259370	
1132.49	-1.29556	0.276684	-4.68248	2.83E-06	0.002263	KCNK6	9424
1678.806	-1.29649	0.246938	-5.25025	1.52E-07	0.000271	ANKRD33B	651746
28.21735	-2.00201	0.505823	-3.95792	7.56E-05	0.01915	CD300E	342510
16.95251	-2.31499	0.609205	-3.80002	0.000145	0.028527	CCR7	1236
37.25818	-2.54466	0.687161	-3.70315	0.000213	0.036005	ITGA2B	3674

Supplementary Table S2: miRNA raw counts table.

miRNA name	VCM_14	VCM_17	VCM_18	VCM_19	ICM_13	ICM_5	ICM_7
hsa-miR-1-3p	12013900	8228310	9438228	10430851	7847928	11110057	1762018
hsa-miR-143-3p	4075989	3367174	2876046	1954576	2979429	2070468	327460
hsa-let-7a-5p	1592377	1515541	1782506	1104384	1235677	1381467	1613307
hsa-let-7f-5p	1005847	853919	1057845	692808	791837	907027	577257
hsa-miR-26a-5p	1019994	853848	900248	641055	633871	585077	268412
hsa-miR-30d-5p	915208	638704	857424	621000	557039	614614	202637
hsa-miR-30a-5p	545129	468504	610764	416463	387885	438726	87089
hsa-miR-30c-5p	361008	362154	406911	247483	396647	323547	85790
hsa-miR-27b-3p	344572	321332	351249	214646	211652	258712	53436
hsa-miR-24-3p	320939	337700	291915	153200	281164	214794	44226
hsa-miR-133a-3p	296044	233150	220799	183811	213379	234575	89135
hsa-miR-21-5p	169348	236156	242298	85555	255594	274786	32737
hsa-miR-125b-5p	234775	240786	258980	120612	146410	160661	82489
hsa-let-7g-5p	217005	201563	201803	160130	174584	168228	117385
hsa-miR-486-5p	186621	100657	98159	113031	117876	97749	334708
hsa-miR-486-3p	186621	100657	98159	113031	117876	97749	334708
hsa-miR-126-3p	202350	165777	185213	153517	158310	130761	27602
hsa-miR-23b-3p	178013	171600	170297	88880	110999	119130	40012
hsa-miR-30e-5p	178998	129557	154413	132078	111301	132770	23485

Supplementary Table S3: differentially expressed miRNAs.

baseMean	log2FoldChange	lfcSE	stat	P value	P adj	miRNA
253.4688	1.143511	0.272814	4.19154	2.77E-05	0.011609	hsa-miR-494-3p
5120.321	0.990214	0.287046	3.449667	0.000561	0.048661	hsa-miR-218-5p
972.3113	0.823972	0.231523	3.558927	0.000372	0.048661	hsa-miR-487b-3p
1331.771	-1.77934	0.509483	-3.49244	0.000479	0.048661	hsa-miR-106b-3p
98.12613	-2.32649	0.676209	-3.44048	0.000581	0.048661	hsa-miR-193b-5p

Supplementary Table S4: top 30 enriched GO terms categorized by the CC.

ID	Description	setSize	enrichmentScore	NES	pvalue	p.adjust
GO:0062023	collagen-containing extracellular matrix	328	0,5453024	2,28518852	1E-10	1,7975E-08
GO:0031012	extracellular matrix	417	0,52806228	2,2665523	1E-10	1,7975E-08
GO:0030312	external encapsulating structure	418	0,52421527	2,24807263	1E-10	1,7975E-08
GO:0005796	Golgi lumen	60	0,66296548	2,2067721	3,8387E-07	1,38E-05
GO:0043679	axon terminus	73	0,64020874	2,18949407	3,2282E-07	1,2216E-05
GO:0098803	respiratory chain complex	71	0,63691274	2,15219082	1,057E-07	4,7497E-06
GO:0005746	mitochondrial respirasome	75	0,61520315	2,11584985	3,2228E-07	1,2216E-05
GO:0070469	respirasome	81	0,6031969	2,10173118	4,8956E-07	1,5304E-05
GO:1902495	transmembrane transporter complex	247	0,50122372	2,05003144	1,9095E-09	1,9613E-07
GO:1990351	transporter complex	267	0,49201299	2,02094026	7,0388E-10	8,4348E-08
GO:0005581	collagen trimer	68	0,56747154	1,91180425	0,00010117	0,001347
GO:0070069	cytochrome complex	32	0,6463447	1,88725612	0,00088592	0,00804619
GO:0008328	ionotropic glutamate receptor complex	24	0,68109712	1,88021852	0,00158098	0,01291732
GO:0098978	glutamatergic synapse	259	0,4467091	1,82941414	4,5566E-07	1,4892E-05
GO:0098798	mitochondrial protein-containing complex	254	0,4414411	1,80746777	1,3879E-06	3,1487E-05
GO:0005761	mitochondrial ribosome	78	0,48178101	1,6745208	0,00205247	0,01604052
GO:1904115	axon cytoplasm	58	0,50667837	1,67127979	0,00384093	0,02681196
GO:0031253	cell projection membrane	267	0,36965444	1,5183533	0,00089527	0,00804619
GO:0001650	fibrillar center	130	-0,3638774	-1,4795298	0,00745849	0,04302924
GO:0032580	Golgi cisterna membrane	78	-0,4223476	-1,5991899	0,00631283	0,03814224
GO:0099023	vesicle tethering complex	59	-0,4497836	-1,6243273	0,00714635	0,04177421
GO:0009897	external side of plasma membrane	276	-0,3874302	-1,7297517	2,2903E-06	4,3335E-05
GO:0044853	plasma membrane raft	95	-0,4472822	-1,7662898	0,00077921	0,00747001
GO:0005801	cis-Golgi network	60	-0,4986404	-1,8015534	0,00126033	0,01078785
GO:0044194	cytolytic granule	13	-0,7253837	-1,8267259	0,00502798	0,0331662
GO:0000137	Golgi cis cisterna	19	-0,6896805	-1,9356659	0,0013227	0,01118852
GO:0005942	phosphatidylinositol 3-kinase complex	25	-0,6651944	-1,9948116	0,0006424	0,00633663
GO:0042101	T cell receptor complex	14	-0,8164057	-2,1131003	6,1597E-05	0,00086839
GO:0071682	endocytic vesicle lumen	17	-0,7822143	-2,1530626	2,9847E-05	0,0004566
GO:0001772	immunological synapse	42	-0,7007075	-2,3511775	1,4223E-07	6,0155E-06

Supplementary Table S5: top 40 enriched GO terms categorized by the BP.

ID	Description	setSize	enrichmentScore	NES	pvalue	p.adjust
GO:0140448	signaling receptor ligand precursor processing	30	0,69429527	2,0251174	0,00012601	0,00351235
GO:0007268	chemical synaptic transmission	484	0,45267679	1,98001763	1E-10	4,2432E-08
GO:0098916	anterograde trans-synaptic signaling	484	0,45267679	1,98001763	1E-10	4,2432E-08
GO:0099537	trans-synaptic signaling	492	0,45088261	1,97156587	1E-10	4,2432E-08
GO:0030198	extracellular matrix organization	245	0,47284428	1,95417501	1,0719E-07	1,6635E-05
GO:0043062	extracellular structure organization	245	0,47284428	1,95417501	1,0719E-07	1,6635E-05
GO:0099177	regulation of trans-synaptic signaling	327	0,45846076	1,9439441	2,984E-09	7,7882E-07
GO:0032964	collagen biosynthetic process	32	0,64343019	1,90526756	0,0003333	0,00714053
GO:0003071	renal system process involved in regulation of systemic arterial blood pressure	19	0,70771021	1,89265222	0,00222826	0,02924954
GO:0003044	regulation of systemic arterial blood pressure mediated by a chemical signal	37	0,61724599	1,88163987	0,00035947	0,00753317
GO:0010257	NADH dehydrogenase complex assembly	53	0,57590728	1,88024167	0,00019098	0,00464674
GO:0032981	mitochondrial respiratory chain complex I assembly	53	0,57590728	1,88024167	0,00019098	0,00464674
GO:0017158	regulation of calcium ion-dependent exocytosis	29	0,6252529	1,81735001	0,00168537	0,02371916
GO:0010712	regulation of collagen metabolic process	29	0,61892282	1,79895111	0,0021348	0,02844082
GO:0035637	multicellular organismal signaling	126	0,46804524	1,78411539	9,5534E-05	0,00290243
GO:0007156	homophilic cell adhesion via plasma membrane adhesion molecules	125	0,45845174	1,7452975	0,00058239	0,01075271
GO:0015909	long-chain fatty acid transport	43	0,55220412	1,73580131	0,00374126	0,04229127
GO:0006813	potassium ion transport	152	0,42967961	1,6670012	0,0006011	0,01093827
GO:0030178	negative regulation of Wnt signaling pathway	145	0,42484151	1,64194267	0,00061891	0,01117544
GO:0071805	potassium ion transmembrane transport	135	0,42747759	1,63744522	0,00119857	0,01865094
GO:0044770	cell cycle phase transition	443	-0,3553325	-1,6470128	7,63334E-08	1,28914E-05
GO:1901857	positive regulation of cellular respiration	11	-0,7529459	-1,8289622	0,003681202	0,041856358
GO:0002263	cell activation involved in immune response	219	-0,4199459	-1,8412835	4,25423E-07	4,77574E-05
GO:0006968	cellular defense response	35	-0,5761738	-1,8812999	0,00137521	0,020890097
GO:0007259	receptor signaling pathway via JAK-STAT	106	-0,47771	-1,8855337	2,0901E-05	0,000902358
GO:0002228	natural killer cell mediated immunity	51	-0,5517357	-1,9222175	0,000100021	0,002991268
GO:0019221	cytokine-mediated signaling pathway	344	-0,4294078	-1,9634103	1,10847E-10	4,24322E-08

Supplementary Table S5 (continued)

ID	Description	setSize	enrichmentScore	NES	pvalue	p.adjust
GO:0050868	negative regulation of T cell activation	96	-0,5079571	-1,9844703	1,77213E-05	0,000770876
GO:0002269	leukocyte activation involved in inflammatory response	37	-0,600168	-1,9900152	0,000263388	0,006001474
GO:0080164	regulation of nitric oxide metabolic process	50	-0,5837799	-2,0300391	5,24989E-05	0,001872353
GO:2001057	reactive nitrogen species metabolic process	67	-0,5465749	-2,0311903	1,09966E-05	0,000517559
GO:0046209	nitric oxide metabolic process	66	-0,551051	-2,0402017	2,81383E-05	0,001152075
GO:1990266	neutrophil migration	81	-0,5417037	-2,0678527	3,11655E-06	0,000198836
GO:0045428	regulation of nitric oxide biosynthetic process	48	-0,6043816	-2,0889318	4,02774E-05	0,001492083
GO:1990868	response to chemokine	59	-0,5940426	-2,1309035	3,07291E-06	0,000198254
GO:1990868	response to chemokine	59	-0,5940426	-2,1309035	3,07291E-06	0,000198254
GO:0030098	lymphocyte differentiation	296	-0,4774399	-2,1622774	1E-10	4,24322E-08
GO:0002367	cytokine production involved in immune response	82	-0,5633417	-2,1623849	6,72138E-07	5,93757E-05
GO:0030217	T cell differentiation	212	-0,5121204	-2,2386641	1E-10	4,24322E-08
GO:0032611	interleukin-1 beta production	71	-0,6243411	-2,3319627	2,28258E-08	4,51951E-06

Supplementary Table S6: top 26 enriched GO terms categorized by the MF

ID	Description	setSize	enrichmentScore	NES	pvalue	p.adjust
GO:0005201	extracellular matrix structural constituent	132	0,60006653	2,27480172	5,1222E-10	1,0808E-07
GO:0015453	oxidoreduction-driven active transmembrane transporter activity	58	0,61004176	2,01121009	1,4327E-05	0,00071977
GO:0048306	calcium-dependent protein binding	63	0,58515261	1,97433487	2,2617E-05	0,00108459
GO:0008324	cation transmembrane transporter activity	418	0,45202681	1,95308215	1E-10	1,055E-07
GO:0016655	oxidoreductase activity, acting on NAD(P)H, quinone or similar compound as acceptor	47	0,60582346	1,93994063	0,00013	0,00457166
GO:0005539	glycosaminoglycan binding	166	0,49620288	1,92154624	5,1626E-07	3,8904E-05
GO:0005518	collagen binding	61	0,57049976	1,90851202	0,00012145	0,00441822
GO:0008137	NADH dehydrogenase (ubiquinone) activity	33	0,6298049	1,87489398	0,00044182	0,01081153
GO:0033293	monocarboxylic acid binding	53	0,5740638	1,87069613	0,00032394	0,00876305
GO:0003954	NADH dehydrogenase activity	36	0,61018152	1,84202274	0,00091843	0,01605471
GO:0015318	inorganic molecular entity transmembrane transporter activity	465	0,40861049	1,77275041	3,2962E-08	3,1613E-06
GO:0016616	oxidoreductase activity, acting on the CH-OH group of donors, NAD or NADP as acceptor	97	0,48606021	1,76771112	0,00046116	0,01081153
GO:0009055	electron transfer activity	104	0,47559756	1,73832136	0,00044388	0,01081153
GO:0004364	glutathione transferase activity	19	0,66463652	1,72904474	0,00443476	0,04742471
GO:0004714	transmembrane receptor protein tyrosine kinase activity	51	0,51412493	1,66915862	0,00411155	0,0456598
GO:0030545	signaling receptor regulator activity	258	0,35136486	1,44370091	0,00207789	0,02846984
GO:0003779	actin binding	384	-0,2891084	-1,360626	0,00228368	0,02902747
GO:0005126	cytokine receptor binding	154	-0,3630985	-1,5003371	0,00225972	0,02902747
GO:0008013	beta-catenin binding	77	-0,4324924	-1,6036433	0,00454456	0,04794509
GO:0005125	cytokine activity	98	-0,4128865	-1,606756	0,00237188	0,02978973
GO:0008094	ATP-dependent activity, acting on DNA	92	-0,4469948	-1,7084209	0,00069551	0,01358814
GO:0019825	oxygen binding	16	-0,6772916	-1,7756399	0,00213555	0,0288847
GO:0016493	C-C chemokine receptor activity	15	-0,7462194	-1,9403621	0,00040353	0,01038345
GO:0019956	chemokine binding	26	-0,6576714	-1,962243	0,00027722	0,00790444
GO:0046935	1-phosphatidylinositol-3-kinase regulator activity	13	-0,8196509	-2,0198798	2,9172E-05	0,0013292
GO:0140375	immune receptor activity	103	-0,5308933	-2,0948165	8,1377E-07	5,0502E-05
